# The Influence of Different Factors on Brain Weight

**DOI:** 10.15388/Amed.2025.32.2.2

**Published:** 2025-12-30

**Authors:** Eglė Skukauskaitė, Greta Asadauskaitė, Sigitas Laima, Sigitas Chmieliauskas, Jurgita Stasiūnienė, Diana Vasiljevaitė, Paulius Petreikis

**Affiliations:** 1 Faculty of Medicine, Vilnius University, Vilnius, Lithuania; 2 Faculty of Medicine, Vilnius University, Vilnius, Lithuania; 3 Department of Pathology, Forensic Medicine, Institute of Biomedical Sciences of the Faculty of Medicine of Vilnius University, Vilnius, Lithuania; 4 Department of Pathology, Forensic Medicine, Institute of Biomedical Sciences of the Faculty of Medicine of Vilnius University, Vilnius, Lithuania; 5 Department of Pathology, Forensic Medicine, Institute of Biomedical Sciences of the Faculty of Medicine of Vilnius University, Vilnius, Lithuania; 6 Department of Pathology, Forensic Medicine, Institute of Biomedical Sciences of the Faculty of Medicine of Vilnius University, Vilnius, Lithuania; 7 Department of Anatomy, Histology and Anthropology, Institute of Biomedical Sciences, Faculty of Medicine, Vilnius University, Lithuania

**Keywords:** brain weight, traumatic brain injury, alcohol intoxication, drug intoxication, strangulation asphyxia, smegenų svoris, trauminis galvos smegenų sužalojimas, intoksikacija alkoholiu, intoksikacija narkotikais, stranguliacinė asfiksija

## Abstract

**Background:**

A postmortem brain weight examination can provide valuable diagnostic information on probable causes of death. Deviations from normal brain weight can indicate the presence of different factors such as psychoactive substance use, the presence of neurological conditions, tumours, brain oedema or traumatic brain injury. The aim of this study is to analyse these factors and their role in understanding the underlying causes of death.

**Materials and methods:**

This research was designed as a retrospective study. The study sample consisted of 651 autopsy cases from 2013 to 2023. The brain weight was compared between people who died from traumatic brain injury, by hanging, of other sudden causes, and were intoxicated by alcohol or drugs. The collected data were processed by using *R* software. *P* values less than 0.05 were considered significant.

**Results:**

The mean brain weight of the control group was 1274.93 ± 124.74 g. The mean brain weight of males was greater than that of females. The brain weight was lower in children and the elderly, whereas the greatest in adults between the ages 21–30. In the ethyl alcohol-intoxicated group, the mean brain weight was 1344.01 ± 148.69 g, whereas, in the drug-intoxicated group, it measured 1418.45 ± 125.45 g. The mean brain weight of subjects with strangulation asphyxia was 1372.13 ± 128.83 g, while for those with traumatic brain injury it was 1358.27 ± 150.42 g. The highest brain weight was observed in subjects with epidural hematoma and with subarachnoid haemorrhage. The most frequent complications in patients who died after brain injury were cerebral herniation and pneumonia. The mean brain weight of subjects with cerebral herniation was 1376.95 ± 164.29 g. After traumatic brain injury, skull fractures, brain surgery and cerebral herniation were associated with a higher brain weight. There was a negative correlation between the brain weight and the *Glasgow Coma Scale* score.

**Conclusions:**

A greater brain mass was observed in subjects with ethyl alcohol and drug intoxication, in the groups with strangulation asphyxia and traumatic brain injury compared to the control group. In the traumatic brain injury group, a greater brain weight was observed in men, in those with skull fractures, with epidural haemorrhage, with herniation signs, and after brain surgery.

## Abstract (it)

**Įvadas:**

Autopsijos metu nustatomas smegenų svoris gali suteikti vertingos informacijos apie tikėtinas mirties priežastis. Smegenų svorio pakitimus gali lemti įvairūs veiksniai, pavyzdžiui, psichoaktyviųjų medžiagų vartojimas, neurologinės ligos, navikai, smegenų edema arba smegenų traumos. Šio tyrimo tikslas – išanalizuoti šiuos veiksnius bei jų sąsajas su pagrindinėmis mirties priežastimis.

**Medžiaga ir metodai:**

Retrospektyvinio tyrimo metu nagrinėtas 651 autopsijos atvejis nuo 2013 m. iki 2023 m. Smegenų svoris buvo lyginamas žmonių, kurie mirė dėl patirtos galvos smegenų traumos, pasikorę, dėl kitų staigių priežasčių ir kurių kraujyje ar šlapime buvo aptikta etilo alkoholio, narkotikų. Surinkti duomenys buvo apdoroti naudojant R programinę įrangą. Statistiškai reikšmingais duomenys laikyti, kai p reikšmė buvo mažesnė nei 0,05.

**Rezultatai:**

Vidutinis kontrolinės grupės smegenų svoris buvo 1274,93±124,74 g. Vidutinis vyrų smegenų svoris buvo didesnis nei moterų. Smegenų svoris buvo mažesnis vaikų ir pagyvenusių žmonių grupėse, didžiausias – 21–30 metų amžiaus suaugusiųjų. Apsvaigusiųjų nuo etilo alkoholio grupėje vidutinė smegenų masė buvo 1344,01±148,69 g, apsvaigusiųjų nuo narkotikų grupėje – 1418,45±125,45 g. Asmenų, patyrusių stranguliacinę asfiksiją, vidutinė smegenų masė buvo 1372,13±128,83 g, patyrusių galvos smegenų traumą – 1358,27±150,42 g. Didžiausia smegenų masė nustatyta asmenims po epidurinio ir subarachnoidinio kraujavimo. Dažniausios komplikacijos pacientams, mirusiems po patirtos galvos smegenų traumos, buvo smegenų strigimas ir pneumonija. Tiriamųjų, patyrusių smegenų strigimą, vidutinis smegenų svoris buvo 1376,95±164,29 g. Po galvos smegenų traumos kaukolės lūžiai, galvos smegenų operacijos ir smegenų strigimas buvo susiję su didesniu smegenų svoriu. Tarp smegenų svorio ir Glazgo komos skalės balų buvo nustatyta neigiama koreliacija.

**Išvados:**

Lyginant su kontroline grupe, didesnė smegenų masė nustatyta asmenų, apsinuodijusių etilo alkoholiu ir narkotikais bei stranguliacinės asfiksijos ir galvos smegenų traumos grupėse. Galvos traumų grupėje didesnė smegenų masė nustatyta vyrų, asmenų, patyrusių kaukolės lūžius ar epidurinį kraujavimą, esant strigimo požymiams ir po smegenų operacijos.

## Introduction

Postmortem brain weight examination can provide valuable diagnostic information on probable causes of death. Deviations from normal brain weight can indicate psychoactive substance use before death as well as the presence of brain damage of various aetiologies (e.g., hypoxia, infection, metabolic derangements), tumours or *Traumatic Brain Injury* (TBI) [1,2]. TBI refers to brain damage resulting from external mechanical factors, excluding degenerative or congenital causes [3]. TBI stands as a leading cause of mortality worldwide, including a substantial proportion of reported injuries, with Europe registering an overall head injury rate ranging from 47 to 694 per 100,000 inhabitants annually in 2021 [4]. In the USA, patients suffering from TBI tend to require extended hospitalization periods compared to individuals without head injuries [5]. Autopsy data on traumatic brain injuries serve as a valuable information source; however, it is not commonly or thoroughly examined in academic literature.

Across numerous countries globally, ethanol stands as the most commonly consumed psychoactive substance among those permitted by law. Additionally, within forensic toxicology laboratories, *Blood Alcohol* (ethanol) *Analysis* (BAC) emerges as the predominant examination conducted, with ethanol being the most prevalent substance detected and documented within the field of forensic toxicology [6]. Systematic analysis of the global burden of disease has shown that alcohol consumption caused 1.78 million deaths in 2020 [7]. Alcohol is linked with brain oedema in animal models. So far, this topic has not been extensively explored in humans [1]. Different factors, such as the mechanism of death (TBI) or alcohol consumption, cause cerebral oedema, which is detectable after death. The aim of this study is to analyse these factors and their role in understanding the underlying causes of death. Analysing these factors could help to understand the underlying causes of death and prevent it.

## Materials and methods

### Study design and data source

A retrospective analysis of data from the State Forensic Medical Service was performed in 651 autopsy cases from 2013 to 2023. The study classified cases into four categories according to the cause and circumstances of death: traumatic brain injury (TBI), intoxication (alcohol or drugs), death by hanging, and controls (those who died of other sudden causes). The TBI group included 71 deaths from brain injury among patients hospitalized between 2013 and 2021. In the TBI group, the brain trauma type, circumstances and complications were analysed. 222 people had drugs and ethyl alcohol in their blood and urine. In 91 of the cases examined, the main cause of death was identified as hanging. The control group included 267 individuals who died from other sudden causes and showed no evidence of traumatic brain injury, no ethyl alcohol or other toxic substances in the blood or urine; cases of hanging due to cerebral stasis were ruled out; also, other cases of asphyxiation (drowning, suffocation) and decomposition (due to autolysis) were excluded. The brain weight was then compared between these groups. Two cases which were excluded from data analysis due to a high brain mass and a possible distortion of the study’s results were described.

In every case, information was provided by the law enforcement agencies, including potential crime scenes, the time of death, and the suspected causes of death. The brain was separated and weighed for each of the deceased.

### Statistical analysis

The collected data were processed by using *R* software. The Shapiro-Wilk test was used to determine whether the data were normally distributed. Student’s t-test was used to assess the statistical significance of differences in continuous variables between the study groups. Spearman’s correlation coefficients were assessed. A weak correlation was defined as r-values <0.39; a moderate correlation with r-values from 0.40 to 0.69 was established; and a strong correlation with r-values > 0.70 was found. Differences with *p* values less than 0.05 were considered significant. When the data followed a normal distribution, the means were evaluated (mean ± standard error), when the data did not follow a normal distribution, the medians were evaluated.

### Toxicology methods

After the forensic dissection, blood and urine samples were collected for alcohol and drug (amphetamine, amitriptyline, barbiturates, benzodiazepines, 3-chloromethcathinone (3-CNC), diphenhydramine, phenothiazine, carfentanil, cocaine, metabolites of cocaine, metamizol, opiates) tests. Headspace gas chromatography was used to detect the presence of alcohol. Meanwhile, liquid chromatography-time-of-flight mass spectrometry (LC/MS-TOF) and chromatography-tandem mass spectrometry (LC-MS/MS) were used for quantitative drug detection. Toxicological tests for alcohol were performed for each case.

### Histological methods

First, histological sections were prepared for routine light microscopy. Histomorphological features of the samples were examined by hematoxylin and eosin (H&E) staining. Next, Perl’s Prussian blue reaction was used to detect ferric iron and Masson’s trichrome staining of the collagen fibres. Hematoxylin and eosin staining consisted of several stages: paraffin removal, staining, and dehydration. Sections were then deparaffinated by keeping them sequentially in absolute alcohol, 96% and 70% ethanol, and distilled water for a certain time. The specimens were stained with hematoxylin and continuously irrigated with flowing water. Subsequently, an eosin-floxed solution was used. Finally, the specimens were quickly sequentially dehydrated in 70%, 90%, and absolute alcohol, and enclosed with a covering material. The nucleus and other DNA/RNA-containing structures were dyed in blue-violet colour while the cytoplasm and the matrix were dyed pink.

### Limitations

The population of Lithuania served as the study’s subject; hence, the results cannot accurately reflect the condition of the entire global population. In our study, we only analysed autopsy data. The exclusion criterion was the presence of advanced putrefaction – autolysis. This study mainly analysed adult brain weights, and therefore the results cannot be fully applied to the paediatric population.

## Results

### The control group

The control group consisted mostly of males 70% (n = 187) with females making up the remaining 30% (n = 80). The mean age of control group was 59.38 ± 19.33 years. The mean age of females (64.56 ± 22.38 years) was statistically significantly higher than the mean age of males (57.16 ± 17.47 years) (*p* <0.05). The mean height of the control group was 168.98 ± 17.03 cm. Women (157.9 ± 18.65 cm) were statistically significantly shorter than men (173.73 ± 13.84 cm) (*p* <0.05). The mean brain weight of the control group was 1274.93 ± 124.74 g. The mean brain weight of females (1214.54 ± 150.77 g) was statistically significantly lower than the mean brain weight of males (1300.75 ± 101.77 g) (*p* <0.05).

Spearman’s rank correlation test showed a statistically significant weak negative correlation (r = -0.18, *p* <0.05) between the age at death and the brain weight. There was a statistically significant moderate correlation between the mean height of the subjects and the mean brain weight (r = 0.4; *p* <0.05).

**Fig. 1:**
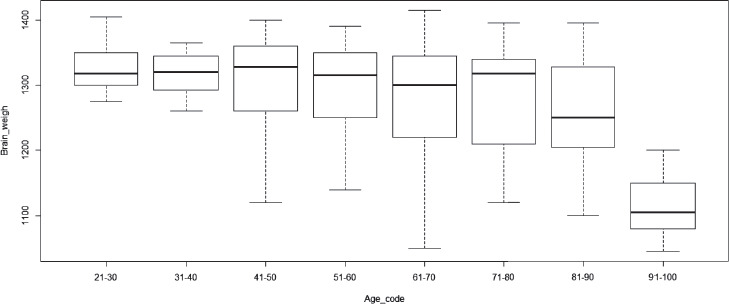
Distribution of median brain weight at different age intervals

The lowest brain weights were established in old individuals (91–100 years old), averaging at 1114.17 ± 54.44 g. The brain weight steadily increased until people reached their twenties (21–30 years old) where it peaked at a mean of 1328.57 ± 43.08 g (Fig. 1). Afterwards, there was a gradual decline of the brain mass, becoming especially pronounced after the age of 90. Overall, the brain weight decreased by about 16% between young adulthood (21–30 years) and a very old age (91–100 years).

**Table 1:** The mean age and brain weight of the control group (male and female)

	Total	Male	Female
*Victims (%)*	100%	70%	30%
*Mean age (years) and SD*	59.38 ± 19.33	57.16 ± 17.47	64.56 ± 22.38
*Mean brain weight (g) and SD*	1274.93 ± 124.74	1300.75 ± 101.77	1214.54 ± 150.77

*Note*. Abbreviations: SD – standard deviation

### The group with detected intoxication

The mean age of the alcohol-intoxicated group was 53.22 ± 14.43 years. The mean age of females (59.78 ± 16.28 years) was statistically significantly higher than the mean age of males (51.69 ± 13.55 years) (*p* <0.05). In the ethyl alcohol-intoxicated group, the mean brain weight was 1344.01 ± 148.69 g. The mean brain weight of the control group (1274.93 ± 124.74 g) was lower compared to individuals with ethyl alcohol detected in their blood and urine with a significant statistical difference (*p* <0.05). The mean brain weight of individuals with drugs detected in their blood was 1418.45 ± 125.45 g. The brain weight of the group with drug intoxication was statistically significantly higher compared against the group with alcohol intoxication (*p* <0.05), and the control group (*p* <0.05).

**Table 2:** The mean age and brain weight of the ethyl alcohol group (male and female)

	Total	Male	Female
*Mean age (years) and SD*	53.22 ± 14.43	51.69 ± 13.55	59.78 ± 16.28
*Mean brain weight (g) and SD*	1344.01 ± 148.69	1375.68 ± 130.19	1238.06 ± 127.04

*Note*. Abbreviations: SD – standard deviation

### The traumatic brain injury group

In the group of subjects with TBI, 63.4% were male (n = 45) and 36.6% were female (n = 26). The median age of the subjects was 58 years (the mean age was 57.90 ± 18.45 years). The mean brain weight of the TBI group (1358.27 ± 150.42 g) was statistically significantly higher than that of the control group (1274.93 ± 124.74 g) (*p* <0.05). When comparing males and females, the mean brain weight of males (1415.53 ± 132.84 g) was statistically significantly (*p* <0.05) higher than mean brain weight of females (1224.54 ± 118.12 g). The mean brain weight of the subjects with a fractured skull (1378 ± 166.66 g) differed from the mean brain weight of the subjects without a skull fracture (1292.78 ± 125.65 g). The mean brain weight of the subjects with subdural hematoma was 1336.63 ± 146.48 g, whereas, in subjects with epidural hematoma, values of 1407 ± 158.75 g were observed, compared with the subjects with brain contusion at 1353.21 ± 147.68 g, and the subjects with subarachnoid haemorrhage at 1364.94 ± 158.48 g. The most frequently observed complications in patients who died after TBI were cerebral herniation (52.1%) and pneumonia (25.4%), while other complications made up 22.5%. The mean brain weight of the subjects with cerebral herniation was 1376.95 ± 164.29 g, and the maximum brain weight was 1750 g. As a result of cerebral oedema, the subjects with TBI, and especially the subjects with skull fractures (1378 ± 166.66 g), those who underwent brain surgery (1386.29 ± 149.67 g), and individuals with cerebral herniation (1376.95 ± 164.29 g) exhibited statistically significantly higher brain weights than the control group (*p* <0.05). There was a statistically significant weak negative correlation (Pearson’s correlation) between the brain weight (oedema) and the Glasgow Coma Scale score (r = -0.3; *p* <0.05). Car accidents (n = 20), falls from one’s height (n = 18) and falls from heights (n = 16) were the most common modes of injury. The patients injured in car accidents were younger than the patients injured in falls (*p* >0.05). The highest number of concomitant injuries occurred in car accidents (n = 17) and falls from height (n = 6).

**Table 3:** The mean age and brain weight of the traumatic brain injury group (male and female)

	Total	Male	Female
*Victims (%)*	100%	63.4%	36.6%
*Mean age (years) and SD*	57.90 ± 18.45	56.16 ± 17.56	60.92 ± 19.89
*Mean brain weight (g) and SD*	1358.27 ± 150.42	1415.53 ± 132.84	1224.54 ± 118.12

*Note*. Abbreviations: SD – standard deviation

### The group of strangulation asphyxia

The mean age of the strangulation asphyxia group was 51.19 ± 18.31 years. The mean age of females (58.70 ± 23.71 years) was not statistically significantly higher than the mean age of males (49.73 ± 16.95 years) (*p* >0.05). The mean brain weight of the subjects with strangulation asphyxia was 1372.13 ± 128.83 g. The mean brain weight of the strangulation asphyxia group was statistically significantly (*p* >0.05) higher compared to the control group. When comparing males and females, there was a statistically significant difference in the mean brain weight of 1398.63 ± 115.18 g and 1237 ± 112.58 g, respectively (*p* <0.05).

**Table 4:** The mean age and brain weight of strangulation asphyxia group (male and female)

	Total	Male	Female
*Mean age (years) and SD*	51.19 ± 18.31	49.73 ± 16.95	58.70 ± 23.71
*Mean brain weight (g) and SD*	1372.13 ± 128.83	1398.63 ± 115.18	1237 ± 112.58

*Note*. Abbreviations: SD – standard deviation

## Exclusions of the study

### Case 1

**Fig. 2:**
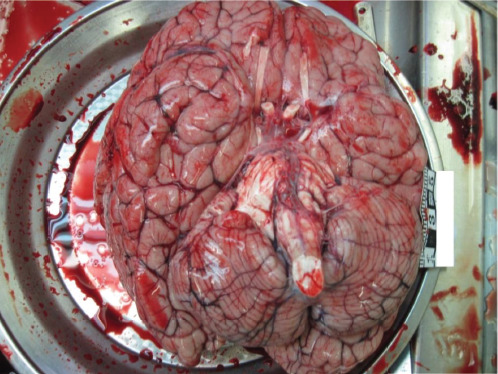
Macroscopic view of the brain (case 1). A photograph displaying the inferior surface of the brain, showing signs of oedema and hyperaemia.

A complete forensic autopsy of a 13-year-old boy of slim build, 166 cm tall, was conducted. He was found dead at home about 1 m away from an electric plug. The investigation found that he connected the electrodes to his chest and plugged the wires into a socket. An external examination of the body showed adhesive electrodes connected to wires taped to the chest. After removing the electrodes, electrical marks were observed in the chest. Internal examination revealed marked oedema and hyperaemia of internal organs. The brain weight was found to be 2000 g (Fig. 2). Toxicology analysis revealed no methyl, ethyl, propyl, butyl and amyl alcohols, acetone or any other toxic substances in the blood of the deceased. The histological findings exhibited morphological features characteristic of electro-marking of the cadaver’s skin, oedema and hyperaemia of the brain and lungs, hyperaemia of the myocardium, liver, pancreas, kidney and adrenal glands. The cause of the death was fatal electrocution.

### Case 2

**Fig. 3:**
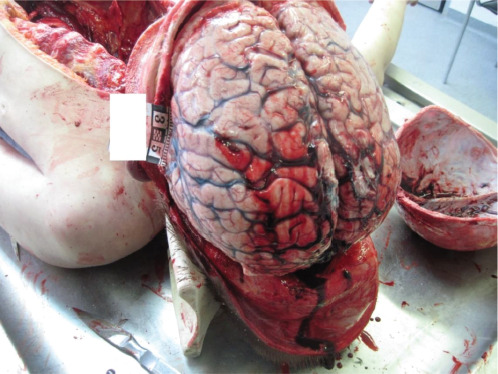
Macroscopic view of the brain (case 2). A photograph displaying the lateral view of the cerebral hemisphere showing the frontal and parietal lobes, with marked hyperaemia, oedema, signs of herniation.

An autopsy of a 10-year-old boy, who was 158 cm tall, with a slim build, was performed. He was found dead at home. He had a fever for a week before his death; the subject ate poorly. An external examination revealed no mechanical injuries on the body. An internal examination revealed oedema and hyperaemia of internal organs. The brain weighed 2229 g, with signs of brain herniation (Fig. 3). Toxicology analysis revealed no ethyl alcohol or other toxic substances in the blood and urine of the deceased. The histological findings exhibited oedema and hyperaemia of the brain, lungs, hyperaemia of the myocardium, liver, pancreas, kidneys and adrenal glands, and also morphological signs of purulent meningitis. The cause of death was purulent meningitis, which was complicated by cerebral oedema, brain herniation, and a progressive respiratory and cardiac failure of central origin.

## Discussion

Molina et al. conducted a prospective investigation involving healthy men between 18 and 35 years old who died suddenly from traumatic causes. The average brain weight in males was found to be 1407 g, ranging from 1070 g to 1767 g [8]. In our study, the average brain weight of males in the TBI group was similar, at around 1416 g.

Three years later, Molina et al. conducted a repeat of their earlier prospective study, this time focusing on healthy females who suffered sudden, traumatic deaths between the ages of 18 and 35. The average brain weight was recorded as 1233 g, with a range spanning from 1000 g to 1618 g [9]. The average brain weight of females in the TBI group was 1225 g, which is also comparable to the results found in literature.

Kandel et al. studied brain oedema in 394 autopsy cases. The study determined the mean brain weight of 1272.38 ± 144.07 grams [10]. In our study, the mean brain weight for the control group was similar at 1275 g. In Kandel et al.’s study, the male brain weight was 1322.47 ± 140.22 grams, whereas the female brain weight was 1221.27 ± 129.55 grams [10]. In our study, we observed comparable results, as the female brain weight was 1215 g, whereas the male brain weighed 1301 g. In Kandel et al.’s study, for both sexes, the maximum brain weight was found in the 21-to-30 years age group [10]. In our study, the maximum brain weight in the control group was also found in the 21–30 years age group (1329 g).

It was observed that women generally have smaller brain weights compared to men [8–10]. The same observation was made by Dekaban et al. who found that, on average, males had brain weights approximately 9.8% greater than females [11]. In our study, both in the control and the TBI groups, the brain weight of females was lower than that of males. In the control group, the male brains weighed about 6.5% more than the female brain. In the TBI group, the brain weight difference was more noticeable, as the male brains weighed 13.5% more than female brains.

Hedman et al. in their study on the brain weight across the lifespan observed the fastest brain growth during childhood and adolescence [11]. This correlated with other studies and our study as well [11,12]. Beyond the age of 35, a consistent decline in the volume is noted, starting at 0.2% per year and gradually accelerating to 0.5% annual volume loss by age 60. Individuals aged over 60 exhibit a continuous volume reduction exceeding 0.5% per year [12]. In Dekaban et al.’s study, a gradual decline in the brain weight was noted around the ages of 45 to 50, reaching its minimum values after the age of 86. By this stage, the mean brain weight had reduced by approximately 11% compared to the peak brain weight [11]. In our study, we observed similar trend with greatest brain mass noticed between the ages 21 and 30. Later on, we observed a gradual brain weight decline which accelerated after the age of 90 years old. The typical decline in the average brain mass from one’s twenties to nineties amounted to approximately 16%.

According to the *World Health Organisation*, alcohol accounts for 5.1% of the global burden of disease and injury, as measured in *Disability-Adjusted Life Years* (DALYs). In 2020, harmful amounts of alcohol were consumed by 1.03 billion males and 312 million females worldwide [7]. Alcohol consumption leads to an early death and disability. In individuals aged 20–39 years, around 13.5% of all deaths are attributable to alcohol [13]. Ethanol is the most commonly legally consumed psychoactive substance globally, with BAC being the predominant analysis conducted in forensic toxicology. Studies in different countries show that 32–46% of subjects have ethyl alcohol in their blood or/and urine at the time of the autopsy [6,14]. Ethanol detection in autopsies is widely reported in the literature, but there is a lack of studies looking at ethanol-induced cerebral oedema. In the present study, a statistically significant correlation was found between blood alcohol levels and increased brain weight detected at the autopsy. In the animal study by Sripathirathan et al., ethanol was associated with neuroinflammation and cerebral oedema [1].

Our study aligns with the existing European data in demonstrating a higher prevalence of traumatic brain injury (TBI) in males (63%), compared to females. Further corroborating this pattern are studies conducted in other European nations, such as Sweden and Ireland, where research indicates a substantial male predominance among TBI patients, ranging from 55% to 80% [4]. This finding is particularly relevant considering the significant burden of TBI in Lithuania. According to the Institute of Hygiene, external causes of death constitute the third leading cause of mortality in the country, following cardiovascular diseases and cancer [15]. A particularly concerning finding in this study was the high prevalence of cerebral herniation, a life-threatening complication arising from brain tissue displacement due to significant swelling. The primary complication identified in the research was cerebral herniation, affecting 52% of the cases, associated with a significant increase in the brain mass. This complication may be more prevalent among younger individuals due to the nature of trauma and brain structural characteristics. Younger patients often sustain head injuries with higher kinetic energy, leading to more severe outcomes. Also, the structural characteristics of the brain differ across age groups. Older brains have enlarged ventricles, and deeper sulci. More space allows for greater swelling before brain oedema or herniation become a risk factor in older patients. In contrast to the literature reporting pneumonia rates of 40–65%, this study observed a lower incidence of this complication at 25% [16]. The risk of pneumonia is amplified by artificial lung ventilation and extended hospital stays [4,16]. This study found a statistically significant increase in the brain mass following skull fractures or brain surgery. The literature suggests that patients with skull fractures have a higher mortality rate [17]. Uncontrolled swelling can lead to a concerning rise in intracranial pressure, potentially causing a fatal brainstem compression. Existing literature suggests that intracranial hypertension is present in a significant amount (45–80%) of TBI patients. Jha et al. emphasise the link between cerebral oedema and poor patient outcomes, highlighting the importance of effective management strategies for this complication [18]. According to the literature, patients with epidural haemorrhage (EDH) are the most frequently operated on (51%) [19]. In our study, both EDH and brain surgery after TBI were associated with a higher brain mass. A higher brain mass in EDH is likely because of higher surgery rates in EDH compared to other brain injury types. In this study, a higher brain mass was correlated with a lower GCS score. Both this study and the existing literature demonstrate that patients with TBI who passed away after arriving at the emergency department had a low mean GCS score [20]. A higher brain mass is likely related to brain oedema and a more severe brain injury, which causes a lower GCS score.

## Conclusion

In the control group, the mean brain weight was found to be statistically significantly related to the age, sex and height. In the group with ethyl alcohol intoxication (1344.01 ± 148.69 g), in the group with drug intoxication (1418.45 ± 125.45 g), and in the group with TBI (1358.27 ± 150.42 g), the mean weights were statistically significantly higher than in the control group (1274.93 ± 124.74 g). In the TBI group, the mean brain weight was statistically significantly higher in men, in those with skull fractures, with epidural haemorrhage, with signs of herniation, and after brain surgery. The brain weight norm is of importance to know in practical work to suspect pathology. In the presence of different factors such as trauma, alcohol and drug intoxication, the brain weight can vary significantly.
